# Rhino-oculo Cerebral Mucormycosis Resistant to Amphotericin B in a Young Patient with Diabetic Ketoacidosis

**DOI:** 10.7759/cureus.4295

**Published:** 2019-03-22

**Authors:** Sarrah Ali Asghar, Zainab Majid, Faryal Tahir, Laila Tul Qadar, Saifullah Mir

**Affiliations:** 1 Internal Medicine, Dow University of Health Sciences, Karachi, PAK; 2 Surgery, Dow University of Health Sciences, Karachi, PAK

**Keywords:** amphotericin b, diabetic ketoacidosis, rhinooccularcerebral mucormycosis

## Abstract

Rhino-oculo cerebral mucormycosis (ROCM) is a rare, invasive, and rapidly progressive fungal infection affecting nose, paranasal sinuses and often extending to orbit, brain, and palate. The immunocompromised, more commonly patients with diabetes mellitus, fall victim to this lethal form of fungus. Although the therapeutic approach includes aggressive surgical and medical interventions, ROCM remains a life-threatening infection with poor prognosis. This rare case addresses the outcomes of ROCM in a young patient with delayed diagnosis and resistance to amphotericin B (Ampho B) contributing to dreadful outcomes.

## Introduction

Mucormycosis (MM) is an opportunistic, life-threatening fungal infection caused by zygomycetes fungi. Six families of mucorales are involved in MM, in which rhizopus is the most common and mucor is the rarest [1]. The population at risk for this lethal infection includes the immunocompromised, such as those having diabetes mellitus, radiotherapy, chemotherapy, a state of neutropenia, hematologic malignancies, excessive iron or aluminum levels, dehydration, diarrhea, metabolic acidosis, and protein energy malnutrition in small children along with chronic hemodialysis, transplantation, steroid therapy, and less commonly, AIDS [2-3]. The most common form of MM in diabetic patients, particularly those with diabetic ketoacidosis (DKA), is rhinocerebral mucormycosis (RCM), an invasive fungal infection with poor prognosis. Currently, amphotericin B (Ampho B), posaconazole, and isavuconazole are the main antifungal drugs used in humans with liposomal Ampho B being the drug of choice. However, the rising resistant strains of fungi pose a severe challenge in the therapeutic approach [4]. Moreover, early diagnosis and prompt medical or surgical therapy remain crucial and extremely important for decreasing mortality, better survival management, and improving the quality of life.

The aim of reporting this case is to highlight the importance of clinical presentation in patients at risk for immunocompromised state for the institution of early investigations and interventions. We report a case of rhino-oculo cerebral mucormycosis (ROCM) in a young patient with uncontrolled diabetes preceding DKA. This case also draws attention to the therapeutic struggles in strains of fungi that are resistant to Ampho B.

## Case presentation

A 16-year-old female was admitted in the ear, nose, and throat (ENT) ward of Dr. Ruth KM Pfau, Civil Hospital Karachi (CHK) with the complaint of ulcers in oral cavity, facial swelling along with oral and nasal discharge for the past one month. According to past history, the patient had a prior episode of DKA one month back which was managed in a local hospital setup in her hometown, Punjab. As part of that management, continuous use of oxygen mask led to the development of ulcer at the nasal bridge, which was not timely addressed. The nasal wound progressed, associated with swelling of the face and erosion of nasal bridge, septum, and palate. A yellowish foul-smelling discharge also appeared, which was occasionally blood tinged. There was no history of ulcers in the past.

**Figure 1:**
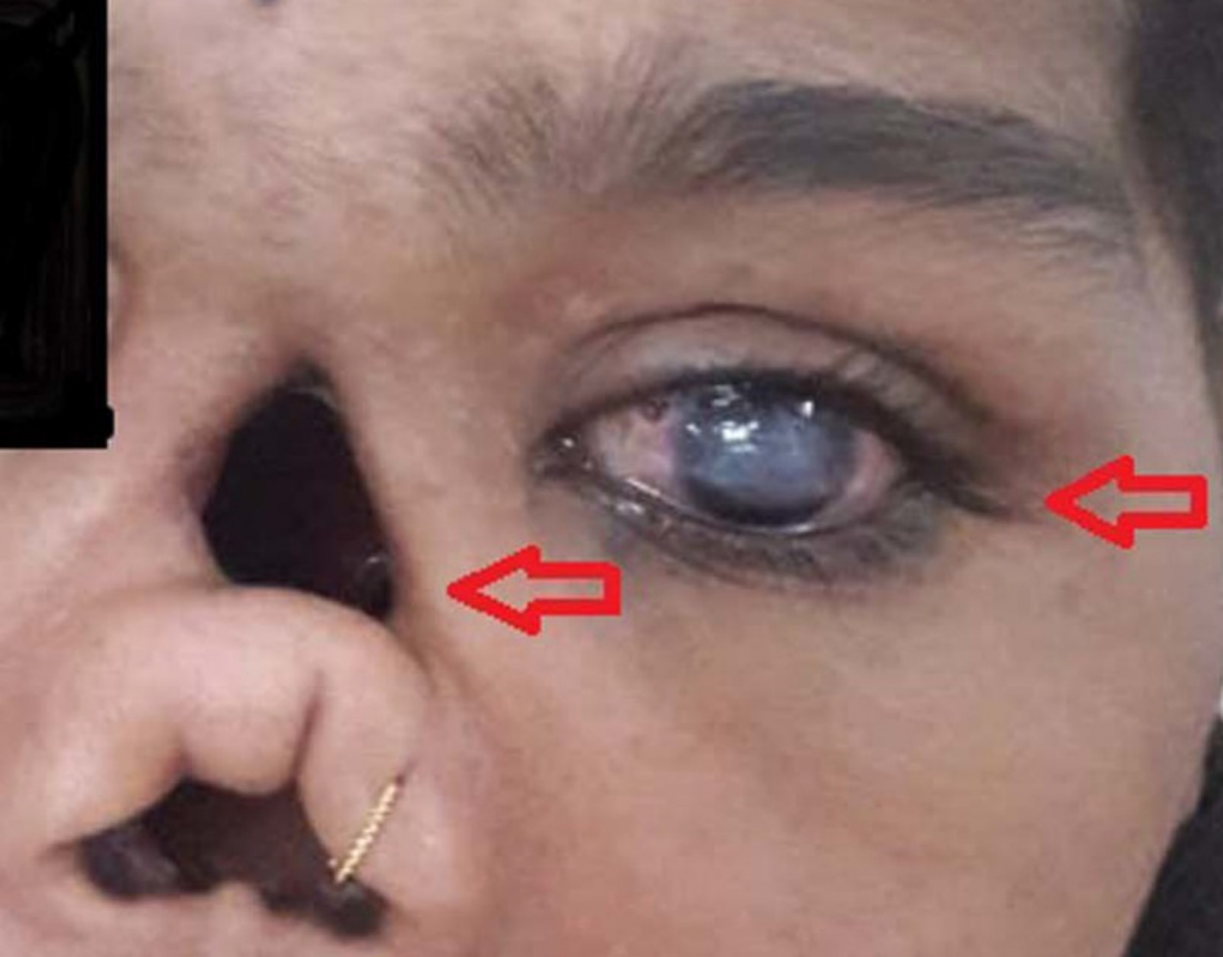
A 16-year-old presenting with nasal septum deformity and haziness of cornea of left eye.

On examination (O/E), the patient was conscious and well oriented to time, place, and person. Upon inspection, a defect was observed over the dorsum of her nose, about 3 cm in diameter, an absent columella, and complete absence of the nasal septum (Figure 1). On eye examination, the left eye revealed decreased vision along with corneal opacities, haziness (Figure 1), and discharge from middle canthal region. Pupillary reflex was also absent in the left eye. The oral cavity inspection showed missing maxillary premolar and molar teeth and a 1 cm oronasal fistula. The rest of the examination was unremarkable.

Laboratory investigations revealed hemoglobin A1c (HbA1c) of 10.5 % [Normal (N) = 4-5.6], random blood sugar (RBS) of 500 mg/dL (N = 79-160), serum potassium (K) of 3.2 mEq/L (N = 3.5-5.0), and a hemoglobin (Hb) of 8.3 g/dL (N = 11.9-15.0).The patient, despite being on an insulin regimen, had her RBS fluctuating in a broad range (60-600 mmol/L). Based on the history, presentation and examination, a working clinical diagnosis of MM infection was made. Treatment with intravenous (IV) injection of Ampho B (50 mg), clindamycin (later replaced by augmentin), and IV vancomycin was shortly started.

**Figure 2:**
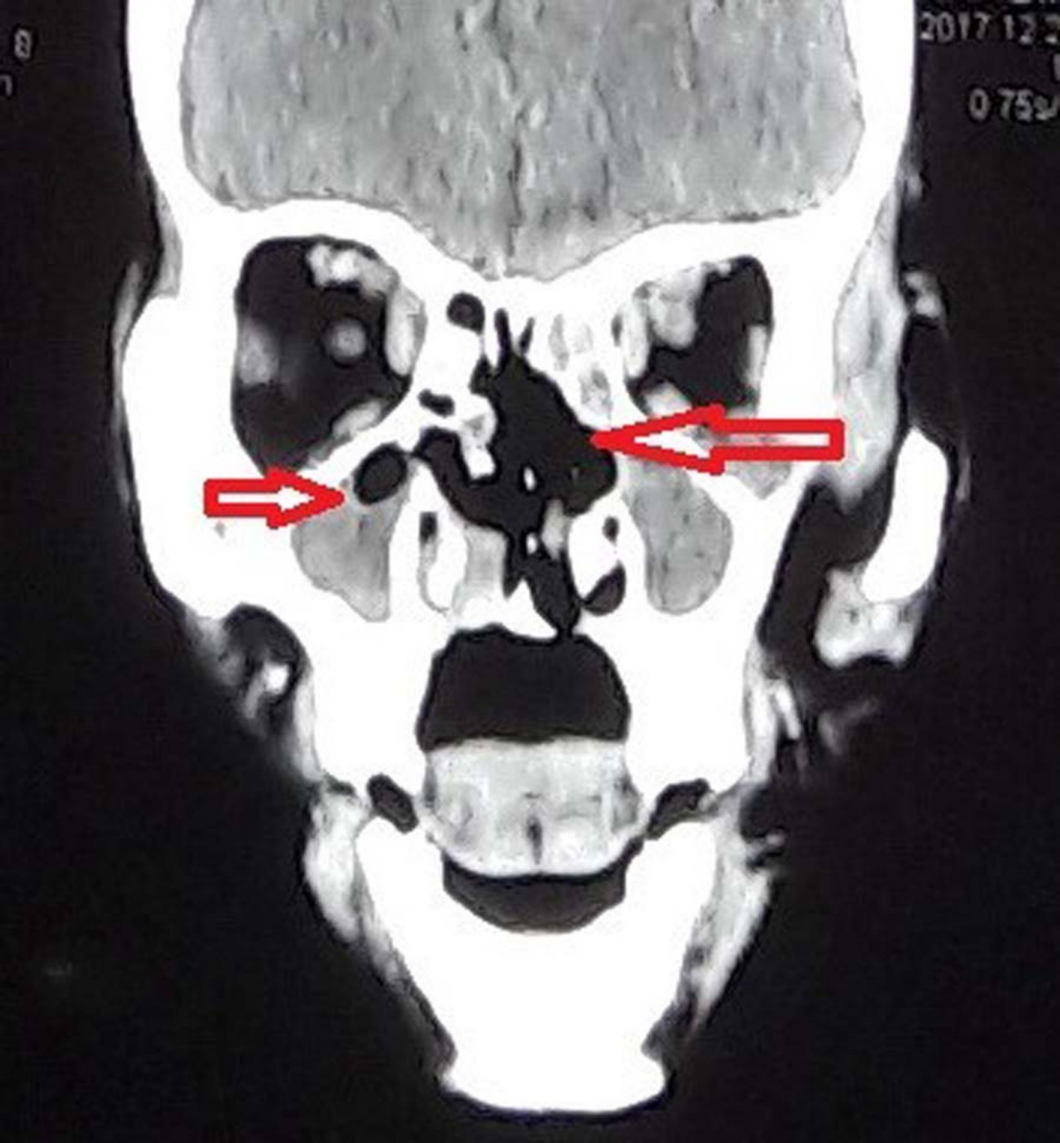
CT scan coronal view showing erosion of the nasal septum, turbinated and the hard palate along with bilateral maxillary sinusitis consistent with invasive fungal sinusitis.

A CT scan of paranasal sinuses was ordered. It revealed opacification of bilateral maxillary and ethmoid sinuses with hyperdense foci extending into the left orbit (Figure 2). Findings were also suggestive of a superimposed fungal infection with intraorbital extension.

**Figure 3:**
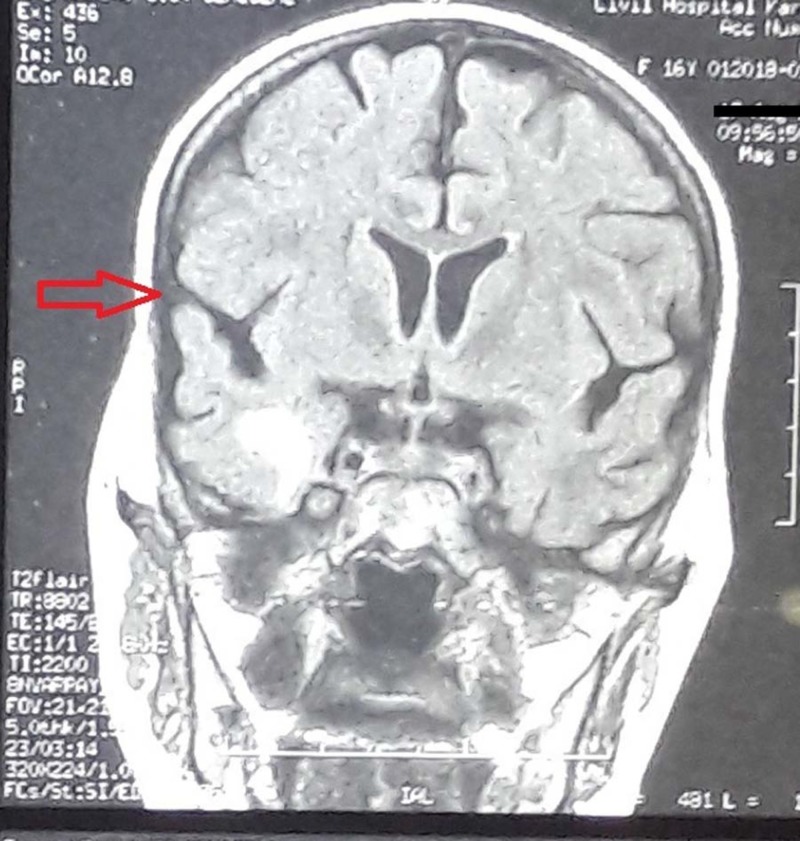
Brain MRI (coronal section) T1 weighted image showing a hypointense signal in the right temporal lobe consistent with intracranial extension of invasive fungal sinusitis.

**Figure 4:**
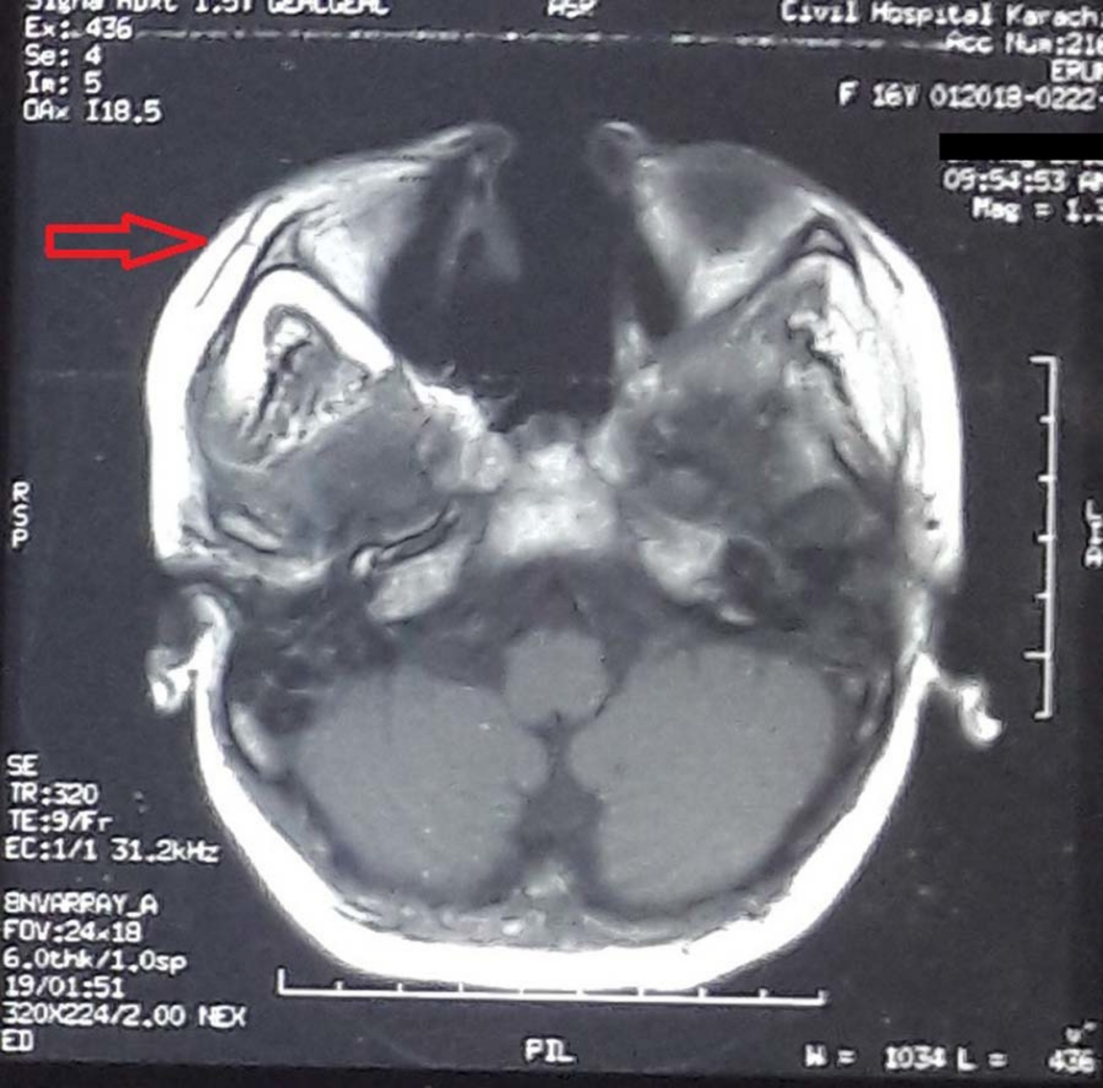
Brain MRI (axial section) T2 weighted axial images hypointense signal signifying intracranial invasion with invasive fungal rhinosinusitis.

The patient underwent a sinonasal debridement procedure in which the infected maxillary and frontal sinuses were surgically removed and the tissue specimen was sent for histopathologic evaluation. The biopsy report later confirmed the presence of MM, which showed broad, nonseptate fungal hyphae having right-angular branches. A brain MRI following the debridement found mucosal thickening over maxillary, left frontal, ethmoid, and sphenoid sinuses. MRI also revealed few hypointense signals invading the cribriform plate and involving the cavernous sinus and temporal lobe posteriorly (Figures 3-4). Three months later, another debridement along with washout of the sinuses was performed. In a second brain MRI, post debridement and medical treatment, a marked improvement was reported in comparison with previous MRI; however, a new onset of left optic canal extension was observed. It was decided to continue Ampho B for a long term as the infection had not resolved. This, however, resulted in hypokalemia, which was K = 2.6 mEq/mol and continued to fluctuate.

Throughout the course of her treatment, repeated expert advice was sought from departments of ophthalmology, dental surgery, and infectious disease. Only to add to the complications, the patient was found partially resistant to Ampho B following the extended treatment duration of nearly eight months. As the infection did not resolve, MM progressed to her temporal lobe and debridement of her infected eye was not preferred keeping in light her quality of life. The patient, after being admitted for an extensive period of 10 months, has been discharged with life-long Ampho B although her prognosis remains poor. The patient is currently followed up in an outpatient department (OPD).

## Discussion

Mucormycosis may manifest in any of the following mentioned systems: rhinocerebral, pulmonary, gastrointestinal, central nervous system, cutaneous, disseminated, and miscellaneous (bones, heart, mediastinum, joints, kidney). The fungal spores in environment, upon inhalation, colonize the paranasal sinus, followed by invasion into the orbit, cavernous sinus and brain, as was evident in our case in which the infection occurred after putting oxygen mask and the patient developed black necrotic tissue in the floor of her mouth and nasal septum. It is a hallmark of this infection due to extensive angioinvasion and vascular thrombosis resulting in tissue necrosis [5]. In our case, debridement of nasal cavity and paranasal sinuses was performed because of the above-mentioned spreading manner of the fungus.

Our patient’s uncontrolled diabetes provided carbohydrate-rich environment for the rapid germination of spores [6]. The acidic environment in DKA favors the growth of mucor by causing dissociation of iron from sequestering proteins, thus delivering free iron to the fungus [7]. As compared to immunocompetent individuals, diabetic patients have reduced phagocytic ability of granulocytes with altered response of polymorphonuclear leukocytes (PMNLs) [6]. After being ignored by the health care staff for a month in her hometown, we started the treatment based on a suspicion for MM according to her history, clinical presentation, and imaging results. However, as the diagnosis was much delayed in our case, it ended up with a worst therapeutic challenge and further survival of the patient is suspected to be only one year. Immediately following her diagnosis, Ampho B was started as it is considered first-line antifungal agent. Regardless of its continued IV infusion for months and surgical debridement of necrotic areas, the infection invaded into temporal lobe as evident by the MRI reports. Even with antifungal therapy and surgical debridement, the overall mortality of patients with MM is high and approaches 45% in RCM [8]. Moreover, our patient also developed partial resistance to Ampho B which is very rare and is often caused by some mutations in the fungal cells that lead to their decreased binding with Ampho B [9]. Therefore, the prognosis of MM infection is generally fair to poor, which largely depends upon the gross health status of the patient, the pace of diagnosis, the ultimate treatment, the ability of the patient to respond to those treatments, and the total debridement of the infected region. In our case, the debridement of the infected eye was not preferred as it was the patient’s only eye providing proper vision.

Apart from the extensive Ampho B therapy and the patient developing resistance to it, another unique feature about our case is the Hb level of the patient, which was decreased (Hb = 8.3 g/dL) in contrary to the usual cases of MM where elevated iron levels support the fungus to grow [7]. Moreover, it might be the first case of MM in our locality with partial resistance to Ampho B. She is one of the rare cases of MM that is neither resolving nor proving fatal despite of all indicators of poor prognosis, which include: delayed treatment of more than six days, intra-cranial invasion, bilateral involvement, and invasion of the palate [8]. Therefore, it is recommended that early diagnosis of this life-threatening fungal infection should be made to save many lives, more importantly, the lives of diabetics with uncontrolled blood sugar levels.

## Conclusions

Early detection of MM is the mainstay factor for good prognosis, however, provided that MM is considered as a differential of any infection in immunocompromised patients and treatment is not delayed. It is essential to stabilize the patient if there are any comorbidities such as DKA, immunosuppression, neutropenia, etc. Rapid and affordable diagnostic methods should be made available nationwide. However, novel therapeutic strategies are needed, and combination therapies should be investigated to combat increasing resistant strains.
